# Chronic Constipation With a Rare Complication of Ischemic Stercoral Perforation of the Rectum: A Case Report

**DOI:** 10.7759/cureus.88474

**Published:** 2025-07-21

**Authors:** Osama M ESayed, Fayruz A Almansouri, Maram M Zahid, Hanan O Alshmaily, Hamad F Alrmaly

**Affiliations:** 1 General Surgery, King Salman Specialist Hospital, Hail, SAU; 2 General Surgery, King Salman Bin Abdulaziz Medical City, Medina, SAU

**Keywords:** abdominal pain, bowel ischemia, chronic constipation, ct imaging, emergency surgery, fecal impaction, fecaloma, hartmann's procedure, rectal perforation, stercoral perforation

## Abstract

Stercoral perforation is a rare but life-threatening complication of chronic constipation, resulting from pressure necrosis of the colonic wall by impacted fecal material. We report the case of a previously healthy 48-year-old woman who presented with acute anal pain and constipation. Despite initial conservative management, her symptoms rapidly worsened. Cross-sectional imaging revealed a large fecaloma in the sigmoid colon with signs of colonic ischemia and perforation. Emergency laparotomy confirmed a substantial anterior rectal wall perforation, and a Hartmann's procedure was performed. Histopathological examination demonstrated transmural necrosis and ulceration consistent with stercoral disease. The patient had an uneventful postoperative course, with resolution of sepsis and stabilization of hematologic abnormalities. This case highlights the importance of early recognition and imaging in patients with severe constipation and abdominal symptoms. Stercoral perforation, though rare, must be considered in the differential diagnosis of acute abdomen, and timely surgical intervention remains essential to prevent catastrophic outcomes.

## Introduction

Fecaloma, defined as a hardened, desiccated mass of fecal material that becomes impacted in the colon, represents an extreme form of chronic constipation [1]. While relatively rare, it is most often seen in elderly, neurologically impaired, or immobile patients with long-standing bowel dysmotility or inadequate colonic evacuation [2]. Unlike simple fecal impaction, fecalomas are often refractory to standard laxatives and can result in serious complications, including bowel obstruction, ischemia, ulceration, and, in rare cases, perforation. Perforation secondary to fecaloma is a life-threatening event associated with significant morbidity and mortality, particularly when the diagnosis is delayed or misattributed to more common causes of acute abdomen [1,2].

Clinical recognition of stercoral perforation is challenging, as initial symptoms of abdominal pain and constipation are nonspecific and overlap with more benign conditions. Radiographic findings, such as colonic distention, pneumatosis intestinalis, and extraluminal air adjacent to a fecaloma, can suggest the diagnosis, but definitive identification often occurs only intraoperatively. Mortality rates for stercoral perforation exceed 30%, largely due to delayed diagnosis, generalized peritonitis, and sepsis [3-5]. Early surgical intervention with fecaloma evacuation, resection of the affected segment, and appropriate stoma formation is critical to improving outcomes.

Despite its high morbidity and mortality, stercoral perforation remains underreported in the literature, with most knowledge derived from isolated case series. Awareness of risk factors, such as chronic constipation, opioid therapy, and immobility, and a high index of suspicion are essential for prompt diagnosis. The present case highlights an otherwise healthy middle‑aged woman without traditional predisposing factors who developed stercoral perforation secondary to a large fecaloma. This report underscores the need to consider stercoral perforation in the differential diagnosis of acute abdominal pain with obstipation, even in the absence of classic risk profiles, and to recognize the radiologic and intraoperative hallmarks that necessitate urgent surgical management.

## Case presentation

A 48-year-old woman, with a long-standing history of constipation, presented to the emergency department with a chief complaint of severe anal pain that had started earlier that day. The pain was associated with constipation and difficulty in defecation but was not accompanied by fever, nausea, vomiting, or diarrhea. On examination, the patient was alert, oriented, and hemodynamically stable, though abdominal distension was noted. Digital rectal examination (DRE) was deferred due to marked pain and increased anal tone. Perianal inspection revealed a posterior midline anal fissure at the 6 o'clock position. Laboratory investigations showed leukocytosis (white blood cell (WBC) count 14.9×10⁹/L) and significant anemia (hemoglobin 7 g/dL). She was discharged on symptomatic treatment, including analgesics, antibiotics (ciprofloxacin and metronidazole), topical lidocaine, lactulose, and sitz baths.

The patient returned later the same day with worsening anal pain, now accompanied by a single episode of non-bilious, non-bloody vomiting. She remained afebrile and denied any diarrhea. On re-evaluation, she appeared tachycardic but remained conscious and oriented. The abdomen was again distended without signs of peritonitis. The fissure persisted, and DRE remained intolerable. General surgery was consulted. The impression was acute anal fissure with associated constipation and symptomatic anemia. The patient was admitted for observation and conservative management, including IV antibiotics, fluids, analgesia, and a high-fiber diet. Further laboratory workup confirmed microcytic hypochromic anemia, and she received one unit of packed red blood cells. Internal medicine was consulted for anemia evaluation, and an iron study was ordered.

**Figure 1:**
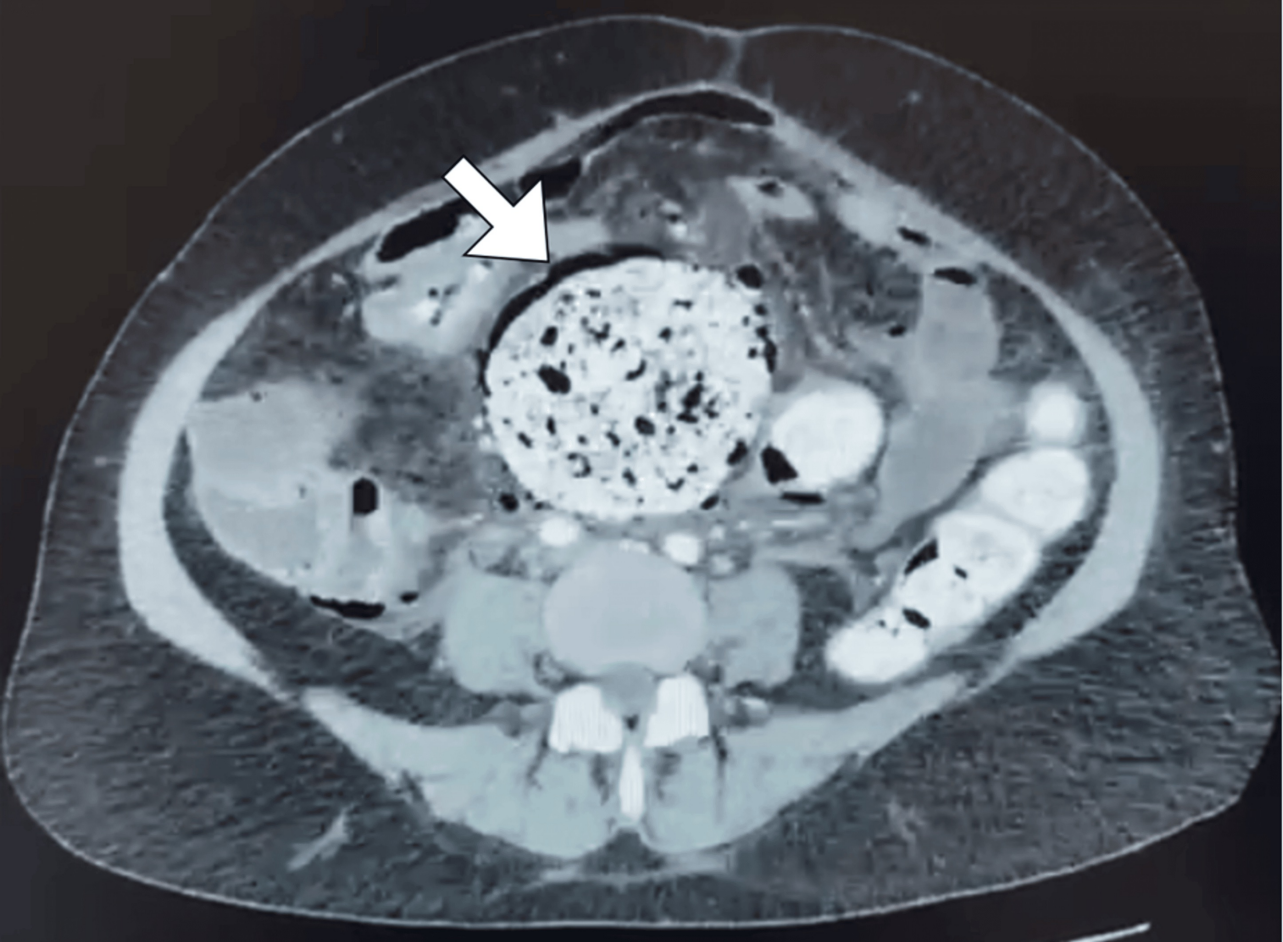
Axial image of contrast-enhanced CT scan of the abdomen The scan demonstrates a distended sigmoid colon (arrow) that is filled with fecal matter and surrounding multiple small air pockets suggestive of perforation. CT: computed tomography

Within 24 hours of admission, the patient's clinical picture deteriorated. She reported new-onset lower abdominal pain, and her WBC count rose to 23.5×10⁹/L with neutrophilic predominance. Despite ongoing analgesia, laxatives, and supportive measures, she remained in significant discomfort. A computed tomography (CT) scan of the abdomen and pelvis with IV contrast was requested due to concern for intra-abdominal complications. The scan revealed a markedly distended sigmoid colon impacted with dense fecal material (fecaloma), with signs of ischemia including pneumatosis intestinalis, mesenteric free air pockets, and localized peritonitis. A collapsed rectum was noted distal to the obstruction, and incidental findings included cholelithiasis with an impacted stone and mild right hydronephrosis. Based on the imaging and clinical findings, fecaloma-induced colonic ischemia and rectal perforation were suspected (Figure 1).

An urgent exploratory laparotomy was performed. Intraoperative findings included a large fecal collection extending from the retrocecal area into the mesentery and presacral region. A substantial perforation was identified in the anterior wall of the upper rectum, involving nearly half of the circumference. The rectum was transected below the site of perforation, and a Hartmann's procedure was performed. The peritoneal cavity was extensively irrigated, and drains were placed in the pelvic and subhepatic regions. The abdominal wall was closed in layers.

Pathological examination of the resected specimen confirmed the presence of a perforated colonic wall with extensive inflammation and surrounding pericolitis. No evidence of inflammatory bowel disease or malignancy was identified. The mucosal surface showed an ulcer measuring 3×2 cm near the area of perforation. The clinical and histological findings supported a diagnosis of stercoral perforation due to fecaloma, a rare but life-threatening complication of chronic constipation.

Postoperatively, the patient was monitored in the surgical ward and continued on IV antibiotics, analgesics, and supportive care. Hematology and internal medicine teams followed her for anemia and electrolyte disturbances, including hypokalemia, which was corrected. She showed clinical improvement and remained afebrile. A colostomy care plan was initiated, and surgical follow-up was arranged for stoma care and eventual consideration of reversal. At the time of discharge, she was stable, with resolution of abdominal pain and normalized inflammatory markers. Further outpatient follow-up was planned with surgery and hematology for anemia evaluation and the long-term management of chronic constipation.

## Discussion

Bowel perforation secondary to fecaloma represents the extreme end of stercoral disease, emerging from chronic constipation and sustained pressure necrosis of the colonic wall. Although most reported cases involve older adults with multiple comorbidities, our patient was relatively young and without a history of opioid use or neurologic impairment, underscoring that stercoral perforation can occur even in the absence of classic risk profiles. In published series, between 69% and 81% of patients with stercoral perforation had documented chronic constipation, and median ages ranged from 58 to 62 years [1-3]. The sigmoid colon is the most common site of perforation, accounting for roughly half of cases, largely owing to its watershed blood supply and the high intraluminal pressures generated by impacted fecal material at that location [2].

The pathogenesis of stercoral perforation involves a cascade beginning with fecal stasis and hardening of stools, forming fecalomas that exert continuous pressure on the rectosigmoid mucosa. As local perfusion diminishes, ischemic ulceration develops and, without relief, progresses to transmural necrosis and perforation. Macroscopic operative findings typically reveal an ulcer crater at the antimesenteric border, with surrounding inflammation. Histopathologically, transmural necrosis and deep ulceration at the site of fecaloma contact are hallmarks, as described in classic series [5,6] and reconfirmed in our specimen. These features distinguish stercoral perforation from other causes of colonic perforation, such as diverticular disease or malignancy, neither of which was evident in our patient.

Early recognition is critical to improving outcomes. Our case illustrates the pivotal role of contrast-enhanced CT imaging, which demonstrated a markedly distended sigmoid colon impacted with dense fecal material, pneumatosis intestinalis, mesenteric air pockets, and localized peritonitis, findings consistent with a diagnostic sensitivity of 90-95% reported in the literature [2-4]. Radiologic criteria include colonic dilation exceeding 6 cm, wall thinning or thickening beyond 3 mm, pericolic fat stranding, and extraluminal air adjacent to a fecaloma. Prompt interpretation of these signs facilitated an expedited surgical decision, likely averting progression to diffuse peritonitis and septic shock.

Although traditional risk factors for stercoral perforation include opioid use, immobility, and institutionalization, emerging data point to other contributors such as chronic kidney disease, diabetes mellitus, and polypharmacy leading to reduced gut motility [7-11]. Our patient's mild renal impairment may have exacerbated colonic hypomotility, promoting fecaloma formation. This observation aligns with epidemiologic studies showing a higher incidence of stercoral complications in patients with multimorbidity, even when constipation is not the sole presenting complaint.

Surgical management remains the cornerstone of treatment. Hartmann's procedure, with resection of the perforated segment, creation of an end colostomy, and peritoneal lavage, yields the best survival outcomes in the acute setting. In our patient, an urgent exploratory laparotomy allowed complete fecaloma evacuation, rectal transection below the perforation, and stoma formation, followed by thorough irrigation and drainage. Postoperatively, multidisciplinary care including broad‑spectrum antibiotics, vigilant hemodynamic monitoring, and correction of electrolyte disturbances was essential. Although reported mortality rates for stercoral perforation range from 17% up to 34-57% in cases with delayed intervention [6,7], our patient's favorable course highlights the impact of early diagnosis and optimized perioperative care on survival.

Despite these advances, stercoral perforation remains underrecognized. Studies have suggested that up to one-third of cases may be missed preoperatively, leading to increased morbidity and mortality [6]. This underscores the need for heightened clinical suspicion in any patient with refractory constipation and acute abdominal symptoms, regardless of age or comorbidity profile. Routine implementation of prompt CT imaging in such scenarios may shorten time to operative management and improve outcomes.

## Conclusions

This case of stercoral perforation due to fecaloma in a relatively young, otherwise healthy woman highlights the need for heightened vigilance in patients with severe, refractory constipation, regardless of traditional risk profiles. Contrast‑enhanced CT played a pivotal role in early diagnosis by revealing characteristic findings, namely, fecal impaction, bowel wall changes, and pneumoperitoneum, enabling prompt surgical intervention with Hartmann's procedure. The favorable outcome achieved through rapid operative management, multidisciplinary perioperative care, and diligent correction of metabolic derangements underscores that timely recognition and aggressive treatment can significantly reduce the high morbidity and mortality historically associated with stercoral perforation. Clinicians should maintain a low threshold for advanced imaging and surgical consultation when faced with obstipation and acute abdominal symptoms to prevent catastrophic complications.
